# Patient-specific record linkage between emergency department and hospital admission data for a cohort of people who inject drugs: methodological considerations for frequent presenters

**DOI:** 10.1186/s12874-020-01163-z

**Published:** 2020-11-27

**Authors:** Rehana Di Rico, Dhanya Nambiar, Belinda Gabbe, Mark Stoové, Paul Dietze

**Affiliations:** 1 Program for Behaviours and Health Risks, Burnet Institute, 85 Commercial Road, Melbourne, Victoria 3004 Australia; 2 Epworth Monash Rehabilitation Medicine Unit, Epworth HealthCare, 32 Erin Street, Richmond, Victoria 3121 Australia; 3 Population Health Research, Turning Point/ Central Clinical School, Monash University, 110 Church Street, Richmond, Victoria 3121 Australia; 4 Department of Epidemiology and Preventive Medicine, Monash University, 553 St Kilda Road, Melbourne, Victoria 3004 Australia; 5 Health Data Research UK, Swansea University Medical School, Swansea University, Swansea, UK

**Keywords:** Record linkage, Data linkage, Administrative data, People who inject drugs, Frequent presenters, Patient pathways, Methods, Australia, VEMD, VAED

## Abstract

**Background:**

People who inject drugs (PWID) have been identified as frequent users of emergency department (ED) and hospital inpatient services. The specific challenges of record linkage in cohorts with numerous administrative health records occurring in close proximity are not well understood. Here, we present a method for patient-specific record linkage of ED and hospital admission data for a cohort of PWID.

**Methods:**

Data from 688 PWID were linked to two state-wide administrative health databases identifying all ED visits and hospital admissions for the cohort between January 2008 and June 2013. We linked patient-specific ED and hospital admissions data, using administrative date-time timestamps and pre-specified linkage criteria, to identify hospital admissions stemming from ED presentations for a given individual. The ability of standalone databases to identify linked ED visits or hospital admissions was examined.

**Results:**

There were 3459 ED visits and 1877 hospital admissions identified during the study period. Thirty-four percent of ED visits were linked to hospital admissions. Most links had hospital admission timestamps in-between or identical to their ED visit timestamps (*n* = 1035, 87%). Allowing 24-h between ED visits and hospital admissions captured more linked records, but increased manual inspection requirements. In linked records (*n* = 1190), the ED ‘departure status’ variable correctly reflected subsequent hospital admission in only 68% of cases. The hospital ‘admission type’ variable was non-specific in identifying if a preceding ED visit had occurred.

**Conclusions:**

Linking ED visits with subsequent hospital admissions in PWID requires access to date and time variables for accurate temporal sorting, especially for same-day presentations. Selecting time-windows to capture linked records requires discretion. Researchers risk under-ascertainment of hospital admissions if using ED data alone.

## Background

Record linkage is increasingly utilised in health services research, longitudinal studies, disease surveillance and health policy [1, 2]; bringing together data from different sources pertaining to the same individual to create richer datasets, while protecting patient privacy [3]. There is growing interest in the use of record linkage to map patient pathways through the hospital system, particularly for groups such as people who inject drugs (PWID), who are frequent users of health services [4], including frequent emergency department (ED) visits [5] and hospital admissions [6]. Mapping these pathways could quantify service utilisation and inform configuration of systems to optimise and maintain positive treatment outcomes [7]. Research in this area is sparse.

Currently in Australia, and many countries internationally, data on patient pathways is not readily available as states and territories collect and store administrative health data from the various hospital sectors in separate databases without common patient identifiers; preventing routine tracking of a patient’s healthcare journey [8]. Mapping pathways therefore requires linkage of patient-specific records within and between multiple administrative datasets, presenting numerous methodological challenges. The Australian Institute of Health and Welfare identified this as a notable gap in health services research [9], impacting our estimates of lengths of hospital stay [10], total hospital costs [11] and disease incidence [12].

Even in countries with established routine record linkage services and unique patient identifiers, such as the United Kingdom [13] and Canada [14], there is no standardised technique to identify which specific hospital admission stemmed from an ED presentation or track patient transfers within and between hospitals to describe continuous episodes of care [15]. Administrative databases are not curated with the goal of data integration and researchers face restricted data access, variable data quality, lack of sufficient computational power and insufficient analyst experience [16–19]. The 2017 GUidance for Information about Linking Datasets (GUILD) publication called for increased transparency and consistency in linkage techniques to improve comparability and interpretation of linked output [20].

The most common methodology for linking ED visits with hospital admissions for a given individual is based on the temporal proximity of these encounters. Administrative timestamps marking the commencement and completion of ED or inpatient encounters are used, though approaches vary. Crilley et al., [21] linked 30% of ED records to hospital records over 2 months at a teaching hospital, where hospital admission timestamps matched ED *departure* timestamps. Ferris et al., [22] linked 23% of ED records to hospital records for patients following drug and alcohol-related ambulance call outs, where hospital admission timestamps matched ED *arrival* timestamps. The proportion of linked records identified has been shown to vary based on the time-lags allowed between ED visit and hospital admission times [23], as well as the clinical aetiology of the ED presentation, which may impact likelihood of admission [24]. For example, Boyle [17] identified hospital admissions for 96% of patients transported to ED following a major road trauma.

These methodological variances require specific consideration in PWID, who often have complex clinical needs, frequent hospital contacts, discharges against medical advice and high risk of re-presentation [25]. Such behaviours generate numerous administrative health records in proximity, including multiple same-day presentations, increasing the complexity of delineating truly linked records. Understanding the challenges encountered in record linkage within a cohort of PWID will assist in optimising future linkage efforts, with potential relevance to other populations who frequently present to hospitals.

In this study, we present a method for patient-specific record linkage between state-wide administrative databases recording ED visits and hospital admission(s) for a cohort of PWID, who are identified as frequent presenters in each dataset. We explore the impact of varying the selected time-windows on linkage results. We present a method to identify continuous episodes of care for an individual patient, demonstrating the yield achieved by including this method within record linkage algorithms for a cohort of PWID. Finally, we comment on the implications of inferring patient pathways using admission or discharge disposition variables from single, stand-alone databases.

## Methods

### Study population

We used identifiable cohort data from 688 PWID recruited between 2008 and 2010 as part of an ongoing longitudinal cohort study; The Melbourne Injecting Drug User Cohort Study (MIX). Participants resided in urban Melbourne, the second largest city in Australia at the time of recruitment, were aged 18 years and over and regularly (at least monthly) injected either heroin or methamphetamine in the 6 months prior to baseline recruitment. Detailed contact information including full name, residential address, telephone number and valid Medicare number (needed to access the universal healthcare system in Australia) were collected at baseline and participants consented to use of this information for data linkage. Contact details and survey data were entered into two separate databases with a unique identifier assigned to each participant, to protect participant confidentiality. Further details on recruitment and baseline characteristics of the MIX cohort are available elsewhere [26].

### Administrative data sources

We accessed administrative data from two, separately stored databases in Victoria, Australia managed by the Department of Health and Human Services; the Victorian Emergency Minimum Dataset (VEMD) and the Victorian Admitted Episodes Dataset (VAED). The VEMD contains de-identified demographic, administrative and clinical details from presentations to 24-h EDs throughout the state [27]. The VAED contains de-identified data for admitted patients in all 429 Victorian public and private hospitals, including rehabilitation centres, extended care facilities and day procedure centres. The VAED stores admission data by ‘episodes’, defined as care provided by one care-type in one campus. Patients transferred between acute and sub-acute care-types before discharge (e.g. transfers from acute surgery to rehabilitation), or transfers between hospitals for continued care, will therefore generate two (or more) VAED records for their one total hospital stay. In Victoria, there is no standardised approach to identify all ‘continuous episodes of care’ comprising one total hospital stay for a given individual. Full descriptions of these databases are available elsewhere [28, 29].

### Ethics

The original MIX study [26] was approved by the Victorian Department of Human Services (now Department of Health and Human Services) and Monash University Human Research Ethics Committees. Written informed consent, including consent to access linked administrative data from VEMD and VAED, was obtained from all participants. The current study was approved by the Victorian Department of Health and Human Services and Monash University Human Research Ethics Committees.

### Record linkage process

**Fig. 1:**
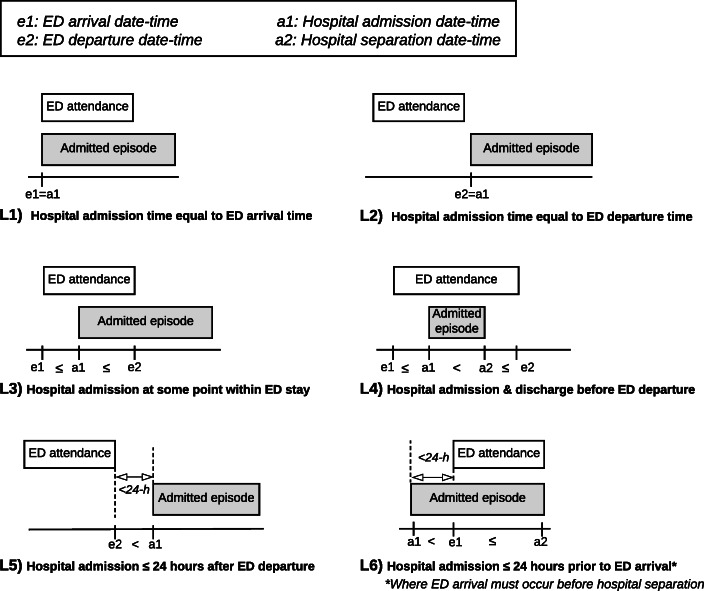
Pre-specified linkage criteria based on temporal relationships between emergency department (ED) arrival/departure dates and times and hospital admission/separation dates and times, used to identify true links

Record linkage occurred in a multi-staged process. Cohort data from the 688 PWID were first submitted to the Centre for Victorian Data Linkage (CVDL), a state-wide data linkage service permitted to receive identifiable patient data. Contact details were uploaded to CVDL using secure data transfer services and record linkage was undertaken subject to all Victorian Privacy Principles that are implemented by CVDL. CVDL deterministically linked cohort data to VEMD and VAED separately, identifying all ED visits and hospital admissions for the cohort between January 2008 and June 2013. CVDL linkage required a 100% match across Medicare number, first three letters of the first name (stored under the variable “Medicare Suffix”), date of birth and sex. CVDL assigned each participant a unique identifier, common between the datasets, and returned de-identified data in Excel format. Two separate datasets were received; one containing clinical and demographic data from ED presentations (‘VEMD data’) and one containing clinical and demographic data from inpatient admissions (‘VAED data’). Combined date-time variables for ED arrivals/departures and hospital admissions/separations were generated to allow temporal sorting of all records; crucial for same-day presentations. Data were interrogated and cleaned for likely coding errors, illogical time entries or discordance with data definitions (*n* = 20 records, < 1%). A multi-way join command merged patient-specific VEMD and VAED data, creating every possible pair-wise combination of an ED presentation and hospital admission for a given individual (*n* = 61,741) [16]. Links were pruned using pre-specified linkage criteria based on temporal proximity of ED visits and hospital admissions, as shown in Fig. 1. In keeping with previous studies [23, 30], linked records were allowed a maximum threshold of 24-h between ED visit and hospital admission. Duplicate joins were reviewed and matched records with the shortest absolute time difference between ED arrival and hospital admission were retained [17]. The resultant dataset included ED visits that required hospital admission (‘linked VEMD and VAED data’), ED presentations that did not require admission (‘unlinked VEMD data’) and hospital admissions without a preceding ED presentation (‘unlinked VAED data’).

### Analysis

Analysis was conducted using Stata 15. The unit of analysis was the administrative health record. There were three main outcomes of interest. The primary outcome was the proportion of linked record-pairs identified, reported as frequencies and proportions, and stratified by the different time-windows selected. The secondary outcome was the proportion of VAED records identified as ‘continuous episodes of care’. Within this study, ‘continuous episodes of care’ (i.e. when a patient was transferred between care-types or campuses before final discharge home, generating two or more VAED records for the one hospital stay) were identified when there was a subsequent, sequential hospital admission occurring within 24-h of an index admission, and the hospital separation mode of the index admission indicated that a transfer of care was planned [11]. Using hospital separation codes that indicated a *planned* transfer of care ensured that unplanned ED visits or re-admissions within 24-h were not included, as these represent distinct clinical pathways (e.g. failed discharges or unrelated/unplanned re-presentations). The tertiary outcome of interest was the performance of key variables within stand-alone datasets in indicating if hospital admissions or ED visits had occurred. Links found were cross-checked against whether links were expected based on VEMD ‘departure status’ codes or VAED ‘admission type’ codes, with frequencies and proportions reported. A summary of key variables and data definitions is presented in Table 1.

**Table 1:** Summary of key variables and data definitions used in the record linkage process

Application	Datasources	
	Victorian Emergency Minimum Dataset (VEMD)^**a**^	Victorian Admitted Episode Dataset (VAED)^**a**^
**For linkage to cohort data**	Medicare number Medicare suffix Date of birth Sex	Medicare number Medicare suffix Date of birth Sex
**For VEMD to VAED linkage**	Unique identifier^b^ Arrival date Arrival time Departure date Departure time	Unique identifier^b^ Admission date Admission time Separation date Separation time
**Useful for manual inspection of links**	Type of visit Referred by Departure status Reason for transfer Campus code ICD-10-AM diagnoses^c^	Admission type Admission source Separation mode Accommodation type on separation Campus code ICD-10-AM diagnosis^c^ Care type Clinical speciality
**For identifying continuous episodes of care**	*Departure status indicating planned transfer of care* Transfer to mental health bed, different hospital Transfer to another hospital campus Transfer to intensive care, different hospital	*Separation mode indicating transfer of care* Statistical separation^d^ Separation and transfer to other hospital
**Cross checking expected versus found links**	*Departure status suggesting admission occurred* Ward setting at this hospital^e^ Procedure room at this campus Transfers to another hospital campus *Departure status suggesting no admission* Return to usual residence^f^ Left before treatment completed^g^ Dead on arrival or died in ED	*Admission type suggesting preceding ED visit* Emergency admission through this hospital *Admission type cannot confirm prior ED visit* Admission from waiting list Other admission^h^ Maternity^i^ Statistical admission^d^ Other emergency admission^j^

^a^Data definitions for VEMD and VAED between 2008 and 2013 were reviewed and amalgamated for this study

^b^Where a unique identifier is assigned by the Centre for Victorian Data Linkage

^c^International Classification of Diseases, 10th revision, Australian Modification

^d^Refers to change in care type within the same hospital

^e^Includes intensive care, mental health beds, other wards, coronary care, mental health observation units, short stay units, emergency medical units and medical assessment and planning units

^f^Where usual residence includes home, correctional/custodial facility, mental health residential facility, residential care home

^g^Includes left at risk after treatment started, left after clinical advice, left at own risk without treatment

^h^Includes planned admissions from outpatient departments, day-surgeries or day treatments (e.g. chemotherapy or dialysis), and follow up admissions following a previous emergency department presentation

^i^Pertains to the admission of a pregnant female of 20 or more weeks’ gestation, or a female within 42 days of giving birth

^j^Includes patients referred from general practice or outpatient clinics for direct ward admission as well as patients that have presented to non-VEMD reporting emergency departments

### Data display and validation

One VEMD encounter was linked to a maximum of one VAED record, with data stored in wide format. For individuals with continuous episodes of care, records were stored in long format, under the index admission. Acknowledging the challenges in evaluating linkage quality in the absence of a ‘gold standard’ dataset [31], linked records with increasing time lags between ED visit and hospital admissions were manually inspected using available additional variables (e.g. diagnostic codes, specialist units, care types, campus codes) to make clinical inferences about the plausibility of these records being truly linked and the nature of the clinical pathway captured. The Stata coding for the linkage algorithm was reviewed by two researchers for quality assurance.

## Results

### Overall linkage results

There were 3459 ED visits identified for the cohort between January 2008 and June 2013, contributed by 492 participants. There were 1877 hospital admissions identified, contributed by 420 participants. Linkage yielded a complete dataset of 4146 records, contributed by 523 unique participants. This dataset comprised 1190 linked VEMD-VAED records (29%), 2269 (55%) unlinked VEMD records and 687 (17%) unlinked VAED records. Therefore, 34% of ED visits (*n* = 1190) were linked to a subsequent hospital admission. Two-thirds (*n* = 1190, 63%) of hospital admissions had a preceding ED visit.

### Links identified based on varying hospital admission time point

Table 2 displays the proportions of links identified by the pre-specified linkage criteria. Most linked records had hospital admission times occurring at some point during the ED stay (L3 = 67% and L4 = 2%), whereas matches with timestamps identical to ED arrival or departure were less common (L1 = 17% or L2 = < 1%).

**Table 2:** Proportion of links identified by varying time-based linkage rule regarding hospital admission time, relative to ED visit

Link	Description	Number of links (***N*** = 1190)	Percentage
L1	Hospital admission time equal to ED arrival time	206	17%
L2	Hospital admission time equal to ED departure time	7	1%
L3	Hospital admission at some point within ED stay	801	67%
L4	Hospital admission and discharge before ED departure	21	2%
L5	Hospital admission ≤24 h after ED departure	43	4%
L6	Hospital admission ≤24 h prior to ED arrival^a^	112	9%

^a^Where ED arrival must occur before hospital separation

When hospital admissions occurred after the patient had departed ED (L5, *n* = 43), most occurred within 2 h (*n* = 30, 70%). When hospital admission times occurred prior to ED arrival (L6, *n* = 112), hospital arrival times were within 11 min of ED arrival in 90% of cases (*n* = 101). In six of the remaining 11 cases, data interrogation suggested likely date entry errors, particularly for encounters spanning the midnight date-change.

### VAED records representing continuous episodes of care

Of the 1877 VAED records in the complete dataset, 1758 (94%) represented index hospital admissions (i.e. initial episodes of admitted care) while 119 (6%) were identified as continuous episodes of care, resulting from planned transfers of admitted patients to another hospital or ward and flowing on sequentially from an index admission. The number of VAED records comprising a total hospital stay varied. Following an index hospital admission (*n* = 1758), the majority of hospital stays were completed in that episode of care (*n* = 1654, 94%), generating only one VAED record. The remaining 104 hospital stays comprised multiple episodes of care, where patients required transfer to a second (*n* = 94), third (*n* = 6), fourth (*n* = 3) or even fifth (*n* = 1) care-type, generating up to five sequentially linked VAED records before final discharge. Therefore, these 104 hospital stays generated 223 VAED records (i.e. 104 index admissions and 119 continuous episodes of care) and involved 63 (12%) of the 523 participants.

### Inferring patient pathways from using stand-alone databases

Table 3 describes the proportion of expected versus found links based on VEMD ‘departure status’ or VAED ‘admission type’ codes. Using the presence of a linked VAED record (*n* = 1190) as the gold standard, VEMD ‘departure status’ accurately indicated that a hospital admission had occurred in 68% (*n* = 813) of cases. In the 32% (*n* = 377) where ED departure status suggested a patient had been discharged to a private residence/facility or left at risk despite the presence of a linked record, the hospital admission time was equal to or in-between the ED visit time in 90% (*n* = 338) of these cases, reducing the likelihood of these being false links. Within the VAED, ‘admission type’ variable, the ‘admitted through emergency department at this hospital’ option was the only code explicitly stating a preceding ED visit had occurred. For records with this code (*n* = 1174), a linked preceding ED record was found in 93% of cases. The remaining options within the ‘admission type’ variable were non-specific regarding a preceding ED visit (*n* = 703) and could therefore not be used to infer patient pathways prior to hospital admission.

**Table 3:** Expected versus found links based on VEMD departure status or VAED admission type

	**Linked VAED record found**		
**Using VEMD departure status**	Yes, *n (col%)*	No, *n (col%)*	
Linked admission expected^a^	813 (68)	59 (3)	**872**
Linked admission not expected^b^	377 (32)	2210 (97)	**2587**
	**1190**	**2269**	**3459**
	**Linked VEMD record found**		
**Using VAED admission type**	Yes, *n (col%)*	No, *n (col%)*	
Preceding ED visit expected^c^	1096 (92)	79 (11)	**1174**
Preceding ED visit uncertain^d^	94 (8)	608 (89)	**703**
	**1190**	**687**	**1877**

*VEMD* Victorian Emergency Minimum Dataset, *VAED* Victorian Admitted Episode Dataset, *ED* Emergency Department

^a^Departure status: Ward setting at this hospital, Procedure room at this campus, Transfers to another hospital campus

^b^Departure status: Return to usual residence, Left before treatment completed, Dead on arrival or died in ED

^c^Admission type: Emergency admission through this hospital

^d^Admission type: Admission from waiting list, Other admission, Maternity, Statistical admission, Other emergency admission

## Discussion

This study presents a method for patient-specific record linkage between separate administrative databases to match ED visits and hospital admissions for a cohort of PWID with frequent hospital contacts. Thirty-four percent of ED records were linked to hospital admissions. Using an array of linkage criteria increased the yield of matched records, but broadening the time-threshold between ED visit and hospital admission increased manual inspection requirements. The majority of hospital stays only generated one VAED record. ED ‘departure status’ coding correctly identified 68% of cases with subsequent hospital admissions.

The proportion of ED records linked to hospital admissions in this study (34%) is comparable to the 36% reported by Wong et al., [23] using similar methods with numerous linkage criteria. It is slightly higher than the 30% reported by Crilley et al., [21] and 25% by Ferris et al., [22] potentially reflecting their narrower linkage criteria requiring identical timestamps. A key methodological consideration is to ensure ED arrival/discharge and hospital admission/separation *dates and times* are requested. A special request may be required as many standard data releases only provide month and year of presentation, which is of insufficient precision to delineate true links in a cohort of frequent presenters who can have multiple hospital contacts in 1 day.

Linkage results must also be interpreted in context of the population or disease in question. The high rate of unlinked VEMD data (*n* = 2269, 66%) in our cohort of PWID likely reflects high-frequency ED usage patterns, use of ED services for presentations not requiring admission [32] and higher rates of leaving before treatment completion. Within our cohort, approximately one third (37%) of hospital admissions were unlinked, with no preceding ED visit. These may represent direct ward admissions, transfers of care, missing data from non-VEMD reporting EDs or failure of VEMD record extraction during the first stage of data linkage. In contrast, Boyle’s [17] study reported only 3.7% unlinked VAED data for victims of major road traumas, whose care pathways more predictably require ambulance retrieval, transportation to ED and direct hospital admission on a single day. Predictable care pathways and single-day events may make record linkage more straightforward, typically generating 1:1 ratios of ED records and admitted episodes for the given event. Requesting timestamps (hh:mm), in addition to datestamps, may be less essential in these cohorts compared to PWID who may have more erratic hospital contact with multiple same-day presentations. Boyle discussed that unlinked hospital admissions in his cohort were likely due to patients being managed in non-VEMD reporting EDs. In our cohort however, other potential sources of bias resulting from lower socioeconomic status, unreliable provision of personal identifiers or less robust data collection at point of care, may introduce systematic linkage errors for PWID that require further exploration [19, 31].

Researchers must also familiarise themselves with relevant administrative coding practices during their study period that may impact linkage rules. During 2008–2013, VAED admission times were recorded when the decision to admit was made and could include treatment time within the ED [33]. As seen in this study, the majority of links had hospital admission times occurring at some point during the ED stay. This was revised in 2016 [34], and care provided within ED is no longer considered part of admitted care, and episodes of care delivered entirely within EDs are not reported to VAED. The epidemiological impact of this administrative change warrants further study, as rates of hospital admissions and lengths of stay may be artificially altered in time series research.

Selecting time-thresholds to define a ‘linked record’ requires discretion, with trade-offs between sensitivity and specificity. Increasing lag times between ED episodes and hospital episodes will increase the proportion of links identified but may alter the nature of clinical pathways captured (e.g. planned discharges home and subsequent planned admissions, or new and unrelated ED presentations). Clinical interpretation from the researcher is required and time-windows must be selected based on the research question. For our cohort of PWID, when the time between ED departure to hospital admission increased beyond a 2-h window, a corresponding increase in manual interrogation and clinical discretion was required to determine if the hospital admission stemmed directly from an ED visit: however, this level of interrogation may not be feasible with larger datasets. Of note, including links where hospital admission times occur *prior* to ED arrival is uncommon in the literature, however there was a notable proportion in this study (*n* = 112, 9%). The large majority (90%) had no more than an 11-min discrepancy in recorded arrival/admission times, likely representing administrative error rather than false matches. Linkage time rules should therefore be based on the study purpose, coding practices and capacity for data interrogation; narrower windows may fail to capture some direct admissions or planned transfers and broader windows may capture some unplanned re-presentations, planned re-admissions or failed discharges.

The absolute incidence of VAED records representing continuous episodes of care was low (6%, *n* = 119). Previous research in this cohort identified that the majority of hospital admissions were due to mental health, drug use, injury or skin infections [5]; conditions which may not require multiple hospital-based episodes of care. Researchers must decide on the value of increasing the complexity of their linkage algorithm to identify these sequential admissions, as it may be more pertinent for certain disease states than others. For example, hip fractures almost universally require at least two episodes of care, from acute orthopaedics to subacute rehabilitation and failure to capture all VAED episodes within one total hospital stay may overestimate incidence of disease and underestimate hospital costs [10, 11].

From an application perspective, this study demonstrates that linking administrative datasets provides more comprehensive and reliable information on patient pathways than using databases in isolation. There are known limitations within ED administrative data [27] and researchers using ED departure status alone to infer discharge pathways risk under-ascertainment of hospital admissions and cannot describe which hospitals, treating teams or services were used during the admitted component of the patient journey. Similarly, researchers are limited in making inferences about pre-hospital resource utilisation or specific patient pathways using the VAED admission-type variable alone. Aside from the option of ‘emergency admission through emergency department at this hospital’, the remaining options were non-specific (see Table 1) reflecting only the broad nature of hospital presentations; emergency versus planned.

### Limitations

This study is subject to the known limitations in data accuracy and completeness within administrative databases. Although data interrogation and re-coding was feasible on this moderate size dataset (< 5000 records), data re-coding was minimised to present a method reproducible for larger datasets. Whilst this study used Australian databases, we believe the insights and approaches offered are relevant for any researcher interested in patient-specific record linkage between administrative databases or mapping patient pathways. The multi-staged linkage process created opportunities for error and, in the absence of a gold-standard dataset, assessing linkage quality remains a challenge [31]. CVDL reviewed their linkage algorithm to minimise false negatives and linked data was interrogated to remove duplicates. The second stage of linkage was based on the assumption that hospital admissions occurring within 24-h of ED presentations are clinically related. Clinically linked episodes occurring beyond this timeframe will have been missed (false negatives) and clinically unrelated episodes within this timeframe may have been linked (false positives). Exploring numerous time-thresholds, identifying episodes of continuous care, thorough manual inspection and cross-checking ‘expected’ versus ‘found’ links minimised these errors. Finally, ED and hospital admission represent only a component of the patient journey. In the absence of common identifiers, system wide data linkage including ambulance, outpatient, ambulatory and general practice databases will be fraught with methodological challenges. Further methodological studies, such as this, will improve our understanding of the strengths and limitations of linkage studies and assist in our analysis and interpretation of linked data.

## Conclusions

Patient-specific record linkage of administrative data from ED visits and hospital admissions is a multi-staged, challenging process in a cohort of PWID with frequent presentations. Researcher discretion is required in selecting time-thresholds for linkage, taking into account the patient population, the specific disease in question and capacity for manual data interrogation. Changes in administrative data-definitions or reporting criteria will have implications for time series research. Researchers using standalone databases to describe subsequent clinical pathways and sequelae will produce erroneous findings and under-estimate the health system burden associated with some specific conditions and behaviours of people presenting to ED. Sharing and evaluating our linkage methods will enable us to devise high-quality, standardised, reproducible linkage systems to unlock the full potential of administrative data whilst preserving patient confidentiality and data security.

## Data Availability

All data used in this study are protected under the privacy policies of Victorian Department of Health and Human Services ‘Deed of Acknowledgment and Confidentiality’. Signed confidentiality agreements prevent us from sharing the data.

## Abbreviations

CVDL: Centre for Victorian Data Linkage
ED: Emergency Department
MIX: Melbourne Injecting Drug User Cohort Study
PWID: People Who Inject Drugs
VAED: Victorian Admitted Episode Dataset
VEMD: Victorian Emergency Minimum Dataset
